# Caregivers’ knowledge and utilization of long-lasting insecticidal nets among under-five children in Osun State, Southwest, Nigeria

**DOI:** 10.1186/s12936-018-2383-5

**Published:** 2018-06-18

**Authors:** Oluwaseyi K. Israel, Olufunmilayo I. Fawole, Ayo S. Adebowale, IkeOluwapo O. Ajayi, Oyindamola B. Yusuf, Abisola Oladimeji, Olufemi Ajumobi

**Affiliations:** 1 Nigeria Field Epidemiology and Laboratory Training Programme (NFELTP), Abuja, Nigeria; 2 Department of Community Medicine, Ladoke Akintola University of Technology Teaching Hospital, Osogbo, Osun State Nigeria; 3 Department of Epidemiology and Medical Statistics, University of Ibadan, Ibadan, Oyo State Nigeria; 4 National Malaria Programme, Federal Ministry of Health, Abuja, Nigeria

**Keywords:** Long-lasting insecticidal net, Under-five children, Malaria prevention, Caregivers knowledge, Nigeria

## Abstract

**Background:**

Utilization of long-lasting insecticidal nets (LLIN) has been associated with reduction of malaria incidence, especially among children. The 2013 Nigeria Demographic and Health Survey revealed Osun State had the least proportion (5.7%) of under-five children (U5) who slept under LLIN the night before the survey. A study was conducted to assess caregivers’ knowledge about LLIN, utilization of LLIN and factors influencing LLIN use among U5 in Osun State, Nigeria.

**Methods:**

A cross-sectional study was carried out among 1020 mothers/caregivers of U5 selected from six communities in Osun State using a multistage sampling technique. A pre-tested interviewer administered questionnaire was used to collect information on socio-demographic characteristics, mothers’ knowledge about LLIN, ownership and utilization of LLIN and factors influencing use of LLIN in U5. Questions on knowledge about LLIN were scored and categorized into good (scored ≥ 5) and poor (score < 5) knowledge out of a maximum obtainable score of seven. Utilization of LLIN was defined as the proportion of U5 who slept under net the night before the survey. Data were analysed using descriptive statistics, Chi square test and logistic regression at α < 0.05. Transcripts from focus group discussions (FGD) were analysed for emerging themes related to caregivers’ perspectives on utilization and factors affecting use of LLIN among U5.

**Results:**

Majority of the respondents 588 (58.3%) fall between age 25–34 years, with a mean age of 30.0 ± 6.3 years. All were aware of LLIN but only 76.1% had good knowledge and 59.0% reported use of LLIN among their U5. Reported barriers to utilizing LLIN were; heat (96.4%), reactions to the chemical (75.5%) and unpleasant odour (41.3%). These were corroborated at FGD. Those with formal education [adjusted odds ratio (aOR) = 1.4; 95% CI 1.0–2.1] and those with good knowledge of LLIN (aOR = 1.8; 95% CI 1.4–2.5) were more likely to use LLIN than their counterparts without formal education and those with poor knowledge of LLIN respectively.

**Conclusions:**

The level of knowledge of respondents about LLIN was high and the utilization of LLIN among U5 was above average, however, it is still far below the 80% target. Efforts should be made to further improve utilization of LLIN through intensified promotion and health education.

## Background

Malaria occurs mostly in the poor, tropical and subtropical areas of the world. It is a leading cause of death and disease in many developing countries especially in sub-Saharan Africa, where under-five children and pregnant women are the groups most affected [1]. In 2016, there were an estimated 216 million cases of malaria, with the WHO African Region accounting for 90% of the malaria cases and 91% of malaria deaths [2]. In areas with high transmission of malaria, more than two-thirds (70%) of all malaria deaths occur in children under 5 and this accounted for 285,000 deaths among under-5 in 2016 [2]. It is the third leading cause of death in children less than 5 years worldwide, after pneumonia and diarrhoeal disease and accounts for almost one out of five deaths in children less than 5 years [3]. Malaria also contributes to malnutrition in children, which indirectly causes the death of half of all children under the age of five throughout the world [4]. Nigeria bears the greatest malaria burden among countries in the world, with over 300,000 malarial deaths each year, most of them occurring in children less than 5 years of age [5]. In Nigeria, malaria is responsible for 30% of the under-five mortality and 11% of maternal mortality rate [6].

Ownership and use of long-lasting insecticidal net (LLIN) is one of the proven interventions adopted by Roll Back Malaria (RBM) partners in Nigeria to stem the high incidence of malaria [6]. It is an important tool for the control of malaria and other vector–borne diseases [7]. Utilization of LLIN has been associated with the reduction in the number of infective mosquito bites by 70–90%, malaria morbidity by 50%, child mortality by 27%, incidence of the malaria parasitaemia by 40% and malaria anaemia by nearly 50% [1, 8, 9]. There has been a rapid scale-up of LLIN distribution in African countries in the recent years [5]. Despite attaining high levels of LLIN coverage [5] many under-five children do not sleep under the net [10]. The 2013 Nigeria Demographic and Health Survey revealed that Osun State had the lowest proportion (5.7%) of under-five children who slept under a LLIN the night before the survey in south-west zone and was second least in the country [11]. Evidence suggests that when large number of people uses LLINs the burden of malaria is reduced, resulting in a reduction in child mortality [12]. Thus, this study assessed caregivers’ knowledge about LLIN, utilization of LLIN and factors influencing LLIN use among under-five children in Osun State, Nigeria.

## Methods

### Study areas

The study area was Osun State, southwest Nigeria. The state has three senatorial districts, namely; Osun Central, Osun East and Osun West. Each district is comprised of 10 local governments, thus 30 local governments overall in the state. The projected population of Osun State for 2015 was 4,449,319 [13], with an estimated under-five population of 889,864. The prevalence of malaria among children under 5 in Osun State was 33.4% [14]. There were 562 public health facilities in the state; comprising of 512 at primary health care level, 48 at secondary and two at tertiary level [15]. Additionally, there were 345 private and 10 missionary health institutions within the state [16].

### Study design

A cross-sectional study design was adopted. Mothers and caregivers of under-five children who were permanent residents in the selected communities in Osun State participated in the study. Caregivers comprised of grandmothers and those who were not biological mothers of the children but were responsible for the care of the child.

### Sample size determination

The minimum sample size of 1020 was calculated using 5.7%, the proportion of under-five children in Osun State who slept under LLIN the night before the 2013 Nigeria Demographic and Health Survey [17], 1.96 standard normal deviate at 95% confidence interval, absolute precision of 0.015 and 10% adjustment for non-response rate.

### Sampling technique

A four-stage sampling technique was used to select the study respondents. One local government area (LGA) was selected from each of the three Senatorial Districts in Osun state, using simple random sampling (SRS) by balloting. The list of all the wards in the three selected LGAs was obtained from Osun State Ministry of Local Government. The wards were stratified into urban and rural wards. Two wards (one rural, one urban) were then selected from each local government by balloting. The list of the enumeration areas in the selected wards was collected from the Local Government Commission (National Population Commission Division) and two enumeration areas were selected from each ward by balloting. A list of all households having at least one under-five child in each selected enumeration area was generated. Households were selected by systematic random sampling. All consenting mothers with the youngest under-five child in the selected households, who were resident in the study areas for at least 12 months were recruited for the study.

### Data collection

Mixed method of data collection was used

### Quantitative data collection

A pre-tested semi-structured, interviewer-administered questionnaire was used. The questionnaire was adapted from the Nigeria Malaria Indicator Survey [18]. The questionnaire had four sections, namely; mother’s socio-demographic characteristics, knowledge of LLIN, ownership and utilization of LLIN and factors affecting the use of LLIN. The questionnaire was written in English language and translated to Yoruba and back-translated to English. This was done in order to retain the original meaning of the questions and to enable the interviewers to interpret the questions correctly without losing the message. The questionnaire was pre-tested among caregivers of under-five children in Osogbo LGA, Osun State which was not a study site. Seven trained research assistants administered pre-tested questionnaires to collect quantitative data. Availability of LLIN in each household was confirmed by physical inspection of net hanging.

### Qualitative data collection

Six focus group discussions (FGD) were conducted among caregivers of under-five children in the six selected wards. Each group consisted of eight participants. Each session lasted between 45 and 60 min. The sessions were moderated by the principal investigator using the FGD guide. Trained research assistants who are Community Health Extension Workers in Ladoke Akintola University of Technology Teaching Hospital were the note takers. The discussions were audio tape recorded.

### Data processing and analysis

The dependent variables of the study were caregivers’ knowledge on LLIN, source of information of LLIN, ownership and utilization of LLIN by under-five children. The independent variables were age, religion, educational level, employment status, residence and number of under-five children in the family. The questions on knowledge about LLIN were scored and categorized into good and poor knowledge. Seven questions assessed respondent’s knowledge on LLIN. Correct responses to questions were scored 1 point, while incorrect responses were scored zero. Respondents who scored below 5 were regarded as having poor knowledge while respondents who scored at least 5 were regarded as having good knowledge. Utilization of LLIN by under-five children was defined as the proportion of under-five children who slept under the LLIN the night before the study.

Data were analysed using Epi-Info version 3.5.1 statistical software. Summary statistics were presented using frequency tables, charts, means and proportions. The Chi square test was used to compare proportions for categorical variables. Predictors of good knowledge and utilization of LLIN were determined using multivariate analysis. Level of statistical significance was set at p < 5%.

The voice recording from the focus group discussions was transcribed and the notes were translated into English language immediately after each session. The FGD notes were studied carefully and analysed using detailed content analysis comprised of: familiarization, identification of themes, indexing, charting, mapping and interpretation [19]. Related to utilization and factors affecting use of LLIN among under-five children, the key themes identified included knowledge on LLIN and malaria prevention, utilization and barriers to utilization of LLIN. Results are presented in narratives.

## Results

A total of 1020 caregivers were interviewed and 1008 questionnaires were returned properly filled and analysed, giving a response rate of 98.8%. Majority of the respondents 588 (58.3%) fall between age 25 and 34 years. Overall, 838 (83.1%) had at least primary education and 170 (16.9%) had no formal education. Nine hundred and sixty-four (95.6%) of them were employed, 828 (82.1%) were skilled workers, 87 (8.6%) were unskilled workers, 49 (4.9%) were professionals and 44 (4.4%) were unemployed. Four hundred and sixty (45.6%) respondents had two or more under-five children in their household and half (50%) of them resided in rural areas (Table 1).

**Table 1:** Socio-demographic characteristics of mothers/care givers of under-five children in Osun State, Nigeria, in April 2015 (N = 1008)

Characteristics	Frequencies (n)	Percentages (%)
Age group (years)		
≤ 24	169	16.8
25–29	335	33.2
30–34	253	25.1
35–39	151	15.0
≥ 40	100	9.9
Religion		
Christianity	524	52.0
Islam	481	47.7
Traditional	3	0.3
Tribe		
Yoruba	896	88.9
Igbo	106	10.1
Hausa/Fulani	6	0.6
Level of education		
No formal education	170	16.9
Primary	301	29.9
Secondary	442	43.8
Tertiary	95	9.4
Employment status		
Employed	964	95.6
Unemployed	44	4.4
Occupation		
Skilled workers	828	82.1
Unskilled workers	87	8.6
Professionals	49	4.9
Unemployed	44	4.4
Number of under five children in the family		
1	548	54.4
≥ 2	460	45.6
Residence		
Rural	504	50.0
Urban	504	50.0

### Respondents’ knowledge on LLIN

**Fig. 1:**
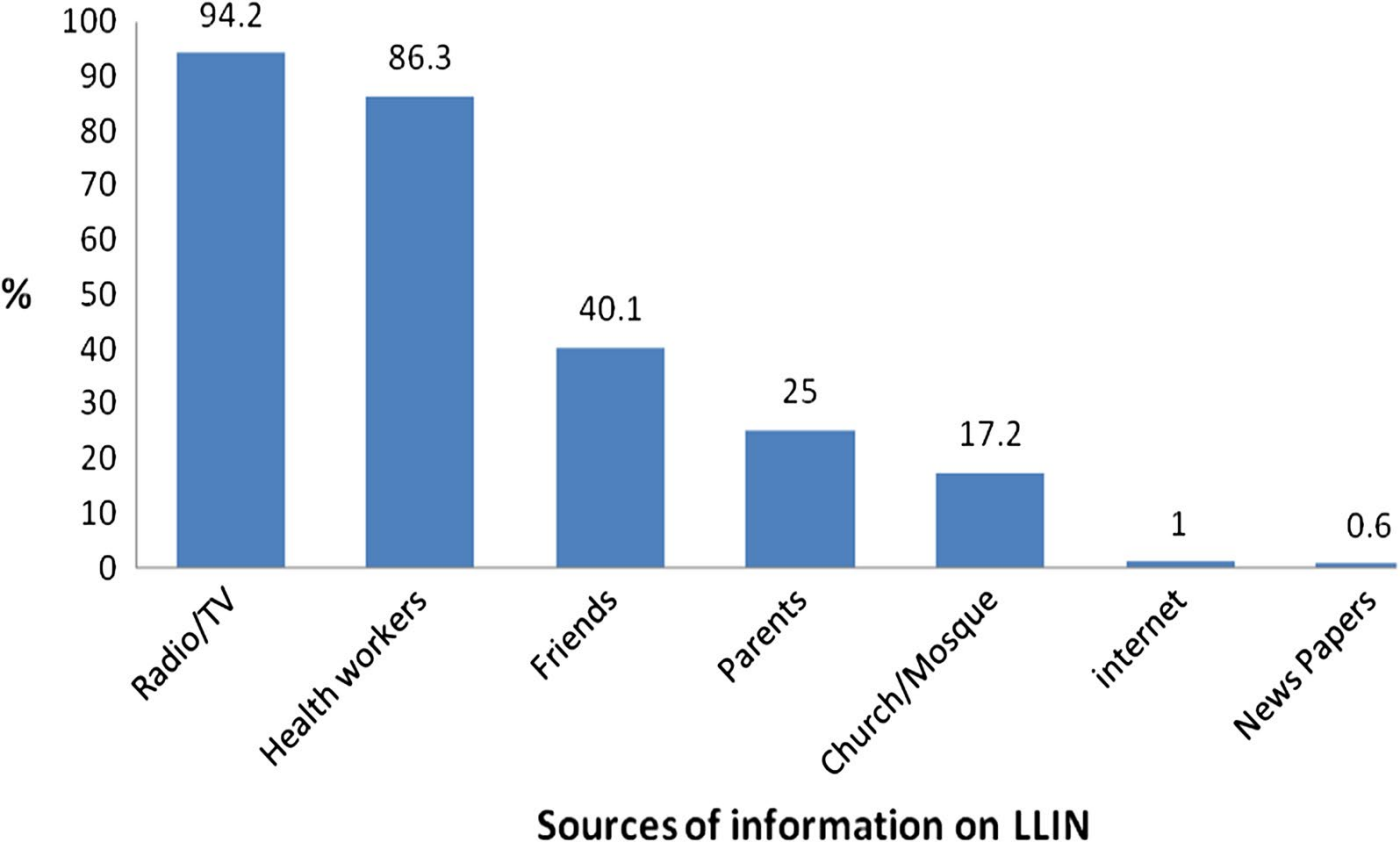
Sources of information on LLIN among mothers/caregivers of under-five children in six selected LGAs in Osun State, Nigeria (N = 1008). Multiple Responses (95% CI 0.8–1.5)

All the caregivers have heard about LLIN and they all gave correct meaning to LLIN, as a bed net treated with insecticide for the purpose of killing and repelling mosquitoes. The main sources of information for the respondents about LLIN were radio and television 950 (94.2%) (Fig. 1). When asked for the advantages of LLIN, all 1008 (100%) respondents said it protects against mosquito bite and malaria infection and 1006 (99.8%) of them also said it protects against other insect bite. Eight Hundred and forty six (83.9%) of the respondents agreed that some people can still come down with malaria despite sleeping under LLIN, 886 (87.9%) of them agreed that the net can offer protection against malaria infection for some years if used consistently, After scoring of the outcome variables for knowledge and categorizing the scores, 767 (76.1%) had good knowledge while 241, (23.9%) had poor knowledge (Table 2).

**Table 2:** Knowledge of mothers/care givers of under five children about long-lasting insecticidal nets (LLIN) (N = 1008)

Variables	Frequency (n)	Percentage (%)
Ever heard about LLINs		
Yes	1008	100.0
No	0	0.0
What is a LLIN		
Correct meaning	1008	100.0
Incorrect meaning	0	0.0
Advantages of LLINs that you know^a^		
Protect against mosquito bite	1008	100.0
Protect against malaria infection	1008	100.0
Protect against other insect bite	1006	99.8
People who sleep under LLINs are protected against mosquito bite, thereby prevent malaria infection in them		
Yes	1008	100.0
No	0	0
Some people who sleep under a LLINs can still have malaria		
Yes	846	83.9
No	162	16.1
LLINs protect a person from malaria infection for some years if used consistently		
Yes	886	87.9
No	122	12.1
Dead mosquitoes on the ground are a good way to tell that your bed net is still effective		
Yes	887	88.0
No	121	121
LLINs only prevent mosquito bites when used with certain types of beds		
Yes	31	3.1
No	977	96.9
More expensive bed nets are more effective than less expensive or free bed nets		
Yes	15	1.5
No	995	98.5
Knowledge categories		
Good knowledge	767	76.1
Poor knowledge	241	23.9
Mean knowledge score	5.79 ± 1.6	

^a^Multiple response

### Ownership and utilization of LLIN by under-five children

Overall, 836 (82.9%) of the respondents had at least one LLIN and 591 (58.6%) of them reported that their under-five children slept under the net the night preceding the survey. Less than half (41.2%) slept every night under an LLIN, while 63 (7.5%) caregivers mentioned their under-five children had never slept under the net. Six hundred and eighty six (82.1%) respondents claimed their net was hung and ready for use, however, 416 (60.6%) LLINs were observed to be hung. Reasons given by caregivers why some under-five children does not sleep under LLIN include excessive heat 402 (96.4%), reactions to chemical 315 (75.5%), unpleasant odour 172 (41.3%), cost is expensive 25 (6%), and no mosquitoes in the house 48 (11.5%) (Table 3).

**Table 3:** Ownership and utilization of LLIN by under-five children in six selected LGAs in Osun State, Nigeria, in April 2015

Characteristics	Frequency	Percentage (%)
Household ownership of LLIN (N = 1008)		
Yes	836	82.9
No	172	17.1
Net claimed to be hanged (N = 836)		
Yes	686	82.1
No	150	17.9
Net observed to be hanged (N = 686)		
Yes	416	60.6
No	270	39.4
Child slept under LLIN the previous night (N = 1008)		
Yes	591	58.6
No	417	41.4
Frequency of child sleeping under net at night (N = 836)		
Every night	344	41.2
Once a week	119	14.2
Twice a week	149	17.8
Three times in a week	77	9.2
Once in a while	84	10.1
Never	63	7.5
Reasons known to caregivers why under five children does not sleep under the LLIN^a^		
Excessive heat	402	96.4
Reactions to chemical	315	75.5
Unpleasant odour	172	41.3
It is expensive	25	6.0
No mosquito in the house	48	11.5

^a^Multiple response

### Determinants of utilization of LLIN

The determinants of LLIN utilization were formal education of caregivers [aOR 1.4, CI 1.0–2.1], and their knowledge about LLIN [aOR 1.8, CI 1.4–2.5] (Table 4).

**Table 4:** Association between utilization of LLIN among under-five children and some selected sociodemographic characteristics in Osun State, Nigeria, in April 2015

Characteristic	Utilization of LLIN			Statistics parameters			
	Yes n (%)	No n (%)	Total (N)	X^2^	*p* value	aOR	95% (CI)
Caregivers’ age (in years)							
≤ 24	103 (60.9)	66 (39.1)	169	1.31	0.86	1.2	1.3–1.6
25–29	196 (58.5)	139 (41.5)	335				
30–34	150 (59.3)	103 (40.7)	253				
35–39	88 (58.3)	63 (41.7)	151				
≥ 40	54 (54.0)	46 (46.0)	100				
Religion							
Christianity	295 (56.3)	229 (43.7)	524	2.84	0.09	1.2	0.6–1.4
Islam	296 (61.5)	188 (38.5)	484				
Caregivers’ educational level							
Formal education	507 (60.5)	331 (39.5)	838	7.17	*0.01	1.4	1.0–2.1
No formal education	84 (49.4)	86 (50.6)	170				
Caregivers’ knowledge level							
Good	476 (62.1)	291 (37.9)	767	15.55	*< 0.001	1.8	1.4–2.5
Poor	115 (47.7)	126 (52.3)	241				
Caregivers’ employment status							
Employed	565 (58.6)	399 (41.4)	964	0.004	0.95	1.1	0.5–2.1
Unemployed	26 (59.1)	18 (40.9)	44				
Residence							
Urban	297 (58.9)	207 (41.1)	504	0.04	0.85	0.9	1.4–1.6
Rural	294 (58.3)	210 (41.7)	504				
Number of under 5 children in the family							
1	313 (57.1)	235 (42.9)	548	1.14	0.29	0.2	0.2–1.4
≥ 2	278 (60.4)	182 (39.6)	460				

* Statistically significant

From the FGDs, most of the participants knew what the LLIN is, and could mention correctly its uses. Most of them said LLIN protects against mosquito bites and malaria and it also protects against other insect bites.

“*LLIN protects against mosquito bites and malaria infection; it also keeps my baby warm during harmattan (female, 38* *years, rural)*”

*“It protects against other insect bites (female, 24* *years, urban)”*

Factors mentioned by caregivers as influencing utilization of LLIN were similar to the survey results. The common themes reported by the participants were the excessive heat, unpleasant odour and adverse reaction to the chemicals.

*“The net causes heat especially during dry season, so some children refuse to sleep under it (female 28* *years, urban)”*

*“It causes body itching, rashes (urticaria), pepperish sensation on the skin and eyes and some children sneeze excessively and may also breath with difficulty when they sleep under the net, in fact there is too much chemical in the net (female, 41* *years, urban)”*

Other factors influencing utilization reported by the participant is the fact that some believe the free nets are more effective than the ones purchased, some also think that LLIN only comes in white colour which get dirty quickly and the lack of awareness by some of the participants that the net comes in different sizes.

*“My child don’t sleep under the net because I didn’t get net during campaign, the free nets are more effective than those purchased (female, 35* *years, rural)”*

*“The net gets dirty quickly because of the white colour and l may not be able to wash it on time (female, 37* *years, rural)”.*

*“I have the net but I don’t use it because the one I bought cannot fit my 7*^*1*^*/*_*2*_ *inch bed (female, 33* *years, rural)”*

## Discussion

This study aimed to assess caregivers’ knowledge about LLIN, utilization of LLIN and the determinants of LLIN utilization among under-five children. The level of awareness of the respondents about LLIN was very high as all respondents in this study have heard about LLINs and all of them could state correctly what it is. This high level of awareness about LLIN have been similarly reported in previous studies conducted among caregivers in Nigeria [20, 21] and other parts of Africa [22]. This pattern is encouraging but may not be surprising in view of the massive investment of the government and non-governmental organizations to reduce the incidence of malaria in recent years. Distribution of respondents according to the knowledge categories of LLINs showed that most of the respondents had good knowledge on LLINs; this suggests exposure to education on LLIN through the mass media, health care providers and community interventions. Community oriented intervention to create awareness are vital to the achievement of the malaria control in the community. A community-based intervention study among mothers in Plateau state showed an increase in the level of awareness of LLIN from 59.2 to 97.6% after health education intervention [23]. Similar to what was reported in previous studies [24–26] and also affirmed by findings from the FGDs, the most common sources of information on LLINs among respondents in this study were radio/television and health workers. This finding is encouraging as this suggests that health workers are frequently in contact with mothers/caregivers. It has also been previously reported that access to health workers and mass media plays an important role in prevention and control of malaria [24].

More than half (58.6%) of the under-five children slept under LLIN the night before the study, this is similar to the findings in previous studies done in malaria endemic countries such as Burkina Faso (70.0%) and Kenya (52.2%) [22, 27]. A high rate (67.5%) was also found in a study done in Southwest Nigeria (6). However, lower use rate was found among under-five children in a Ghanaian study (43.0%) [28], which was adduced to the respondents believe that mosquitoes were few or absent in their environment and their perceived harmlessness of malaria as a health problem [28]. The Nigeria Demographic and Health survey (NDHS) 2013 report showed that only 16.1% of under-five children slept under an LLIN the night before the survey [11]. The 2015 Nigeria Malaria Indicator survey reported that 43% of under-five children slept under an LLIN the night before the survey [29]. Though there seem to be a gradual increase in the rate of utilization of LLIN, the goal is to achieve at least 80% [6] so as to reduce malaria morbidity and mortality. Despite the level of utilization in this study, only two-fifth of all the under-five children sampled slept under the net on regular basis. This is not encouraging as regular use of the net is necessary to reduce the incidence of malaria among under-five children.

The common barriers to utilization of LLIN among under-five children include excessive heat (96.4%), reactions to chemical (75.5%), unpleasant odour (41.3%) and cost 25 (6%). These findings are similar to the barriers to utilization found in previous studies in Enugu, Anambra, Ogun and Ghana [20, 30–32].

The determinants of utilization of LLIN were having formal education by caregivers and their knowledge about LLIN. Previous studies have confirmed our results that formal education influences use of nets in children [8, 33, 34]. Formal education exposes caregivers to knowledge [35] on health issues including LLIN. It was also found in this study that 6 out of 10 respondents with good knowledge of LLINs used the net for their children as compared to 4 out of 10 of those with poor knowledge. This is similar to what was found in a study in Kenya [22], where utilization of LLINs by under-five children was found to be positively associated with knowledge. Several reports confirmed that regular use of insecticide-treated nets increased when individuals received information about bed nets [36, 37]. In a study conducted in malaria endemic area of Iran, regular use of LLINs increased from 58.3% to 92.5% following educational intervention [38]. Therefore, effective educational programmes may further increase use of LLINs in this studied population. However, barriers to use of LLINs among under-five children in this study need to be addressed through health education. There is need for better understanding of proper handling of the net prior to utilization by caregivers to avoid most of the side effects of the chemicals. Some caregivers believed that the free nets were more effective than the net purchased, some also think the net comes only in white colour which makes it difficult to maintain and some were ignorant of the fact that the net comes in different sizes. These misconception and ignorance of caregivers can be corrected through health education.

The strength of this study is the fairly large sample size, being a community based study and the probability sampling technique used which makes the sample to be representative.

The limitations of this study should be noted. The total number of persons in the household was not collected which makes the estimate of ownership difficult. This was probably so because the focus of the study was mainly on under-five children. Also, the information obtained from this study was based on recall, which could lead to recall and social desirability bias. However, the researchers encouraged caregivers to speak the truth and assured that the information obtained will be kept confidential.

## Conclusions

The study revealed that the level of awareness about LLIN among respondents was high, and their knowledge about LLIN was correspondingly high. The utilization of LLIN among under-five children was above average, however, it is still far below the 80% target. The daily use is poor with only two-fifth sleeping under the net on daily basis. Factors identified to influence LLIN use were respondents’ level of education and knowledge of respondents on LLIN. Educative programmes on the use of LLIN should be intensified through the television/radio in other to capture caregivers with poor knowledge on LLIN. Adult education should be encouraged among caregivers in other to improve uptake of LLIN. Improvement of electricity power supply by the government may also encourage utilization of LLIN and amelioration of the discomforting properties of the net by manufacturers also stands to further improve utilization.

## Abbreviations

FGD: focus group discussion
LGA: local government area
LLIN: long lasting insecticidal net
NDHS: Nigeria Demographic and Health Survey
RBM: Roll Back Malaria
SRS: simple random sampling
U5: under-five children
